# Association between sedentary behavior and daily step count with physical performance of community-dwelling older adults participating in an exercise program interrupted by the COVID-19 pandemic: a cross-sectional study in Brazil

**DOI:** 10.1590/1414-431X2026e14959

**Published:** 2026-08-07

**Authors:** V.R.S. Santos, G. Cassemiliano, A.C.S. Farche, S. Lee, L.B. Message, P.G. Rossi, A.C.M. Takahashi

**Affiliations:** 1 Departamento de Fisioterapia, Universidade Federal de São Carlos, São Carlos, SP, Brasil; 2 Laboratório de Pesquisa em Saúde do Idoso, Universidade Federal de São Carlos, São Carlos, SP, Brasil

**Keywords:** Aging, Sedentary behavior, Physical performance, Physical activity, Exercise

## Abstract

One of the effects of the COVID-19 pandemic was the interruption of exercise programs for older adults, significantly impacting their physical performance (PP). Few studies have identified the effects of the interruption of exercise programs on the PP of older adults, and none have assessed the relationship between this outcome and objectively measured daily step count and sedentary behavior (SB). This study assessed the association between SB and daily step count with PP in 42 community-dwelling older adults (73.86±6.78 years old, 88.10% female) after an 18-month interruption of an exercise program due to the COVID-19 pandemic. SB and daily step count were measured using the activPAL3™ micro accelerometer, while PP was assessed through the 30-s sit-to-stand test, handgrip strength test, and Timed Up and Go (TUG) test. Data were analyzed using Pearson correlation test and linear regression model. Results showed that daily step count was significantly correlated with PP (r=0.38 to 0.42). Regression analysis revealed that a mean increment of 1,000 steps/day was associated with a mean increase of 0.31 repetitions in the sit-to-stand test, 0.89 kgF in handgrip strength, and a mean reduction of 0.30-s in TUG time. Additionally, older adults who reached approximately 9,325 steps/day demonstrated better PP outcomes compared to those who accumulated a lower number of daily steps. However, SB was not significantly associated with PP (P>0.05). These findings suggest that accumulating high amounts of daily steps may help mitigate declines in PP when structured exercise programs are interrupted.

## Introduction

Step count is an easily identifiable measure of ambulatory physical activity (PA) and is associated with a lower risk of chronic diseases (1), hospitalizations (2), and mortality (3,4) in older adults. Conversely, sedentary behavior (SB) is inversely related to the benefits of PA (5). Older adults are estimated to spend 65-80% of their time in SB, averaging approximately 9.4 h per day engaged in this behavior (6). Both outcomes can be easily monitored using pedometers and accelerometers (3,7). A higher number of daily steps is associated with improved physical performance (PP) in older adults (3), whereas high levels of SB are linked to poorer PP (5).

A high PP is an important outcome in maintaining an active lifestyle and is related to the development of healthy aging (8). Low levels of PP can triple the likelihood of mortality (9). Moreover, physically active older adults tend to have a lower risk of falls and cognitive impairment, as well as a better quality of life (10). Therefore, any personal or environmental factor that alters active behavior can directly impair the PP of older adults and, consequently, their quality of life (11).

One of the main factors that directly influenced the lifestyle of the global population was the COVID-19 pandemic that was declared in March 2020. Older adults were among the groups most affected by both the direct and indirect consequences of the pandemic (12). Longitudinal studies have shown a decline in PP among community-dwelling older adults (13,14), and cross-sectional studies conducted during the pandemic observed that PP, specifically lower limb strength and handgrip strength, were related to the level of PA and SB (15,16).

Another significant consequence of the COVID-19 pandemic was the abrupt interruption of structured exercise programs for older adults. Although some studies have examined changes in PA, SB, and PP during the pandemic, important gaps remain. For instance, Lefferts et al. (14) assessed changes in PA, SB, and PP over the course of the pandemic but did not investigate associations between these outcomes. Choudhury et al. (16), on the other hand, identified associations between objectively measured PA and SB and PP in older women. However, their sample was limited to females and did not include individuals with prior participation in structured exercise programs. Consequently, studies are needed to investigate the association between PA, SB, and PP after discontinuation of participation in structured exercise programs.

Our study aimed to assess the association between SB time and daily step count, measured objectively, with the PP of Brazilian community-dwelling older adults after an 18-month interruption of an exercise program due to the COVID-19 pandemic. In this study, our hypothesis was that time spent in SB and daily step count are associated with PP in older adults. Additionally, we hypothesized that older adults with higher step counts and lower SB have better PP.

## Material and Methods

### Study design and ethical aspects

This was an observational, descriptive, and cross-sectional study conducted with data collected 18 months after the interruption of a multicomponent exercise program due to the COVID-19 pandemic, in September 2021 (17). This study was developed and structured following the recommendations of the “Strengthening the Reporting of Observational Studies in Epidemiology (STROBE) statement: guidelines for reporting observational studies” (18).

The study was conducted considering the ethical principles established in resolution number 466/2012 of the National Health Council and the Declaration of Helsinki and was approved by the Research Ethics Committee for Human Subjects of the of the Federal University of São Carlos (Ethical Approval Number: 4.126.247/2020). All participating older adults who agreed to participate in the study signed an informed consent form.

### Participants and context

The participants in this study were community-dwelling older adults (≥60 years of age) from São Carlos (a city in the state of São Paulo, Brazil) who had preserved ambulation capacity and were participating in a multicomponent exercise program. A non-probability convenience sampling was used to recruit eligible individuals from the program. The program was conducted three times a week, with each session lasting 50 min, until March 2020 when it was interrupted. This exercise program is offered by the community outreach project “*Revitalização Geriátrica*” (Geriatric Revitalization), which is a partnership between the Federal University of São Carlos and the São Carlos Educational Foundation. Older adults with cognitive impairment, assessed by the Mini-Mental State Examination (MMSE) with a score <18 points (19), and those with physical limitations that would prevent them from performing any of the PP tests used in the study were excluded.

### Measurements

The study data were collected in September 2021, 18 months after the interruption of the multicomponent exercise program. The data were collected at the participants' residences. The variables used were sedentary time, daily step count, and PP, which included lower limb strength, handgrip strength, and functional mobility.

### Sedentary time and daily step count

Sedentary time and daily step count were assessed using the activPAL3™ micro accelerometer (PAL Technologies Ltd., United Kingdom), considered the gold standard equipment for evaluating SB (20). The accelerometer was attached to the middle and anterior third of the participant's thigh using hypoallergenic adhesive tape. Participants wore the device continuously for 7 days and received instructions on its proper care. The accelerometer data were transferred to the PALanalysis software (version 8.11.6.70) and then extracted into a summary spreadsheet (Microsoft Excel) for daily data. The first and last days of assessment were excluded from the analysis as they did not include a full 24 h of use, resulting in a total of 5 consecutive and complete days being considered. Sleep time was obtained using the Primary Lying Time of the activPAL software and was excluded from the analysis (21). Sedentary time was obtained by summing the time spent sitting and lying during wakefulness. The average from the 5 valid days was calculated, with sedentary time is presented in minutes per day and daily step count reported in steps per day (22).

### Physical performance

#### Lower limb strength

The lower limb strength was assessed through the 30-s sit-to-stand test. The participants were instructed to sit on a chair with a height of 42 cm, with their backs supported against the backrest, feet flat on the floor, and arms crossed over their chest. On “go”, the participants stand completely and sit down as many times as possible in 30 s without using their arms. Before starting the test, the researcher demonstrated the procedure and conducted a familiarization round of up to three repetitions (23).

#### Handgrip strength

Handgrip strength was assessed using the Lafayette^®^ hydraulic dynamometer (model J00105; Lafayette Instrument, USA). The participants sit on a chair without armrests with their shoulders adducted and neutrally rotated, elbows flexed at 90 degrees, forearms in a neutral position, and wrists between 0 and 30 degrees of extension and 0 and 15 degrees of ulnar deviation. They are then instructed to squeeze the dynamometer as hard as possible with their dominant hand upon the verbal command and stimulus from the evaluator. Three measurements were taken, with a 1-min interval between each, and the highest value was recorded. The values are reported in kilogram-force (kgF) (19).

#### Functional mobility

The Timed Up and Go (TUG) test was used to assess functional mobility. In this test, participants sit on a chair with armrests with their back against the chair, rise up (using their usual walking aid if necessary), walk at a comfortable safe pace to a line 3 m away, turn around, walk back, and sit down. Timing starts on the word “go” and stops when the person is fully seated again (24).

### Sociodemographic and health characteristics

Data to characterize the study sample were collected during the anamnesis, and included age, gender (women or men), and hip and waist circumference (cm). Systolic and diastolic blood pressure (mm/Hg) were manually assessed using a sphygmomanometer and a stethoscope, and heart rate (bpm) was assessed with a digital pulse oximeter (Incoterm OX520, Brazil). Additionally, the number and type of comorbidities, number and type of medications, number falls in the last year, COVID-19 diagnosis in the last 18 months (yes or no), and cognitive performance via the Mini-Mental State Examination (MMSE) were also collected.

### Statistical analysis

Statistical analyses were performed using IBM^®^ SPSS software (version 26.0, USA), adopting a significance level of P<0.05 and a 95% confidence interval. The normality of the data distribution was assessed using the Shapiro-Wilk test. A descriptive analysis was conducted for the participants' characteristics. Quantitative variables with a normal distribution are reported as mean and standard deviation, while qualitative variables are presented as absolute and relative frequencies. Subsequently, percentiles were calculated for the variables, and participants were categorized into four groups according to the quartiles of the daily step count: Quartile 1: ≤4,896 steps/day; Quartile 2: >4,896 and ≤7,146 steps/day; Quartile 3: >7,146 and ≤9,325 steps/day; and Quartile 4: >9,325 steps/day.

To assess the correlation between sedentary time and daily step count with PP variables (lower limb strength, handgrip strength, and functional mobility), Pearson's correlation test was performed using the mean of the variables. Variables that showed significant correlations were used in linear regression models to examine the effect of sedentary time (per additional 60 min/day) and daily step count (categorized and per 1,000-step increments) on participants' PP. The reference category for daily step count was the first quartile (≤4,896 steps/day). The dependent variables were lower limb strength, handgrip strength, and functional mobility. The independent variables in the model were sedentary time and daily step count. Unadjusted and adjusted regression models were conducted, with the number of comorbidities and COVID-19 infection included as control variables for each PP outcome.

A sensitivity analysis was also conducted to identify the effect of gender on PP variables, daily step count, and sedentary time, given the imbalance in gender distribution. This analysis was performed by comparing mean differences between men and women using the independent samples *t*-test.

## Results

### Participant characteristics

Forty-two older adults were included in this study. The participants were predominantly women (88.10%) and had an average of 0.86±0.90 comorbidities, with systemic arterial hypertension being the most prevalent condition (47.6%). Approximately 76.20% did not report any falls in the past year, and 95.20% had not been diagnosed with COVID-19. Detailed characteristics of the older adults are presented in Table 1.

**Table 1:** Sociodemographic and health characteristics of the participants (n=42).

Variables	Mean±SD or n (%)
Age, years	73.86±6.78
Gender	
Men	5 (11.90%)
Women	37 (88.10%)
Waist-hip ratio	0.93±0.07
Systolic blood pressure, mm/Hg	121.46±14.24
Diastolic blood pressure, mm/Hg	79.29±8.66
Heart rate, bpm	70.64±10.78
Number of comorbidities	0.86±0.90
Systemic arterial hypertension	20 (47.6%)
Hypothyroidism	8 (19%)
Type 2 diabetes	3 (7.1%)
Hypercholesterolemia	3 (7.1%)
Number of medications	2.71±2.26
Antihypertensives	20 (47.6%)
Lipid-lowering agents	20 (47.6%)
Hypoglycemic	6 (14.3%)
Antidepressant	10 (23.8%)
Thyroid hormone replacement	8 (19%)
Falls in the past year	
Yes	10 (23.80%)
No	32 (76.20%)
COVID-19 diagnosis	
Yes	2 (4.80%)
No	40 (95.20%)
Mini-Mental State Examination (0-30)	27.8±1.46

SD: standard deviation.

Table 2 presents the results of all measured PP variables, including the 30-s sit-to-stand, handgrip strength test, and TUG tests, as well as sedentary time and daily step count.

**Table 2:** Summary of correlations, scale ranges, means, standard deviations, and percentiles.

Variables	Lower limb strength	Handgrip strength	Functional mobility	Sedentary time	Daily step count
Lower limb strength	-	0.41*	-0.55*	-0.11	0.38*
Handgrip strength		-	-0.53*	-0.04	0.40*
Functional mobility			-	0.18	-0.42*
Time spent in SB				-	-0.31*
Daily step count					-
Mean	11.31	24.50	10.02	477.03	7593.75
SD	2.67	6.41	2.27	122.77	3257.23
Minimum	5.0	10.0	6.75	219.84	2548
Maximum	15.0	42.0	17.05	815.40	15608.8
25th percentile	9.25	20.0	8.55	391	4896
50th percentile	11.0	24.0	9.45	473	7146
75th percentile	13.0	29.5	10.7	532	9325

SB: sedentary behavior; SD: standard deviation. *P<0.05; Pearson correlation.

### Correlation analysis

Significant correlations (P<0.05) were found between daily step count and lower limb strength (r=0.38), handgrip strength (r=0.40), and functional mobility (r=-0.42). These findings suggest that PP is positively associated with daily step count. No significant correlations (P>0.05) were found between sedentary time and the PP variables.

### Regression analysis

Table 3 presents the results of unadjusted and adjusted regression models for daily step count and the PP variables, controlling for COVID-19 diagnosis and number of comorbidities. The results revealed that older adults who exceeded 9,325 steps/day demonstrated better lower limb strength performance, handgrip strength, and functional mobility compared to those who performed 4,896 steps/day or fewer. In addition, older adults who achieved between 7,146 and 9,325 steps/day also showed better functional mobility performance compared to those who performed 4,896 steps/day or fewer.

**Table 3:** Linear regression analysis between daily step count (1,000 additional steps/day and categories) and physical performance in older adults.

Steps/day	Physical performance variables					
	Lower limb strength		Handgrip strength		Functional mobility	
	β [95%CI]	P	β [95%CI]	P	β [95%CI]	P
Unadjusted						
Quartile 4	2.27 [0.03, 4.51]	0.047*	6.18 [1.0, 11.37]	0.021*	-2.77 [-4.56, -0.98]	0.003*
Quartile 3	2.10 [-0.19, 4.40]	0.072	2.16 [-3.16, 7.47]	0.417	-2.26 [-4.09, -0.43]	0.017*
Quartile 2	0.90 [-1.40, 3.20]	0.432	-0.75 [-6.06, 4.57]	0.778	-1.83 [-3.66, -0.003]	0.050*
Quartile 1	1 [Reference]		1 [Reference]		1 [Reference]	
1000 steps/day increment	0.31 [0.07, 0.55]	0.014*	0.78 [0.20, 1.35]	0.010*	-0.29 [-0.50, -0.09]	0.005*
Adjusted						
Quartile 4	2.29 [1.12, 0.02]	0.048*	7.20 [3.0, 11.40]	0.001*	-2.86 [-4.65, -1.08]	0.003*
Quartile 3	1.87 [-0.48, 4.23]	0.116	1.42 [-3.0, 5.84]	0.339	-2.05 [-3.90, -0.20]	0.031*
Quartile 2	0.72 [-1.68, 3.11]	0.547	2.08 [-2.27, 6.42]	0.519	-1.83 [-3.71, 0.06]	0.058
Quartile 1	1 [Reference]		1 [Reference]		1 [Reference]	
1000 steps/day increment	0.31 [0.07, 0.56]	0.014*	0.89 [0.43, 1.34]	<0.001*	-0.30 [-0.50, -0.10]	0.004*

The table presents the unadjusted regression models and the models adjusted for the control variables COVID-19 diagnosis and number of comorbidities. Quartile 1: ≤4,896 steps/day; Quartile 2: >4,896 and ≤7,146 steps/day; Quartile 3: >7,146 and ≤9,325 steps/day; Quartile 4: >9,325 steps/day. *P<0.05.

Our results also showed that a mean increment of 1,000 steps/day was associated with a mean increase of 0.31 repetitions in the 30-s sit-to-stand test (P=0.014) and a mean increase of 0.69 kgF in handgrip strength (P*<*0.001). Furthermore, a mean increment of 1,000 steps/day was associated with a mean reduction of 0.30-s in the TUG test (P=0.004).

### Effect of gender on physical performance, daily step count, and sedentary behavior

Table 4 presents the results of the sensitivity analysis conducted to identify the effect of gender on PP variables, daily step count, and sedentary time. The results revealed that women had lower handgrip strength (P*<*0.001) and less sedentary time (P=0.022) than men.

**Table 4:** Sensitivity analysis to identify the effect of sex on physical performance variables, daily step count, and sedentary time.

Variables	Women (n=37)	Men (n=5)	*t*	Mean difference [95%CI]	P
Lower limb strength, repetitions	11.3±2.6	11.4±3.6	0.079	-0.10 [-2.71, 2.50]	0.937
Handgrip strength, kgF	23.2±5.4	34.0±5.7	4.184	-10.78 [-15.99, -5.58]	<0.001*
Functional mobility, seconds	10.1±2.4	9.52±1.0	0.521	0.57 [-1.63, 2.77]	0.606
Daily step count, number	7703±3309	6786±3041.3	0.586	917 [-2245, 4079.1]	0.561
Sedentary time, minutes	461.3±118.5	593.3±94.0	2.381	-132 [-244, -19,9]	0.022*

Data are reported as mean±SD or mean difference and 95%CI. *P<0.05; Student's *t*-test.

## Discussion

The results partially confirmed our initial hypothesis. Only the level of PA, measured by daily step count, was correlated and associated with the PP of older adults. Sedentary time was not associated with PP. The mean increment of 1,000 steps/day was positively associated with better results in the PP variables. Additionally, older adults who achieved higher daily step counts (>9,325 steps/day) had better PP than those with lower step counts (≤4,896 steps/day). Our findings also revealed that women had lower handgrip strength and sedentary time than men.

Daily step count showed a significant positive association with lower limb strength and handgrip strength, suggesting that a higher number of steps per day may be linked to increased muscular strength, even during the COVID-19 pandemic, as previously observed in other studies (15,16). Our results revealed that older adults who exceeded 9,325 steps/day performed approximately two additional repetitions in the 30-s sit-to-stand test and exhibited 7 kgF higher handgrip strength compared to those who accumulated 4,896 steps/day or fewer. Consistently, we also found that each mean increment of 1,000 steps/day was associated with increases of 0.31 repetitions in the 30-s sit-to-stand test and 0.89 KgF in handgrip strength. Similar findings were reported in two reviews by Ramsey et al. (3,25), which identified a direct relationship between step count and both outcomes. Together, these findings underscore that older adults who achieve higher step counts tend to perform better on PP measures. Hsueh et al. (26) also demonstrate that accumulating at least 7,000 steps/day is associated with better lower limb performance, both cross-sectionally and prospectively.

Daily step count also showed a significant and negative association with functional mobility, as assessed by the TUG test. Unlike the other variables, a shorter time to complete the TUG test indicates better performance (24). Therefore, our findings showed that a mean increment of 1,000 steps/day was associated with a mean reduction of 0.30-s in TUG completion time, indicating better functional mobility. Similar findings were reported in other studies conducted during the pandemic, in which older adults with higher levels of PA showed better functional mobility (27). Conversely, it is important to note that older adults who achieved approximately 7,146 steps/day or more showed TUG completion times that were 2 to 2.8 s shorter compared to those who achieved fewer than 7,146 steps/day. Our findings may offer important clinical insights, indicating that increasing daily step counts can help improve performance in key variables essential for maintaining a healthy and active lifestyle. However, it is important to emphasize that older adults should be encouraged to gradually increase their step counts until they reach the recommended 7,000 steps/day to achieve greater health benefits.

Consistent with our results, several studies conducted prior to the COVID-19 pandemic identified that higher step counts are associated with better PP (3,28,29). A greater number of daily steps has also been linked to other health benefits in older adults, such as lower incidence of chronic diseases, fewer hospitalizations, and reduced risk of all-cause mortality (3,4). These findings reinforce the recommendations of health organizations that older adults should be encouraged to increase their levels of PA (30). Moreover, our results indicated that accumulating steps throughout the day is a feasible, cost-effective, and easily understandable strategy to recommend to older adults (1). It is important to note that PA recommendations based on step count suggest accumulating at least 7,000 steps/day to achieve better health outcomes in the older population (1).

On the other hand, we did not observe a significant negative association between sedentary time and PP, which contrasted with recent evidence reporting such relationships (3). Physiologically, SB has been linked to poorer PP in older adults because prolonged sedentary time reduces energy expenditure and muscular contractile stimulation, suppressing lipoprotein lipase activity, decreasing insulin secretion, impairing glucose uptake in skeletal muscle, and increasing pro-inflammatory cytokines (31-33). However, the absence of an association in our study may be partly explained by the characteristics of our sample. The participants of this study had previously been engaged in an exercise program before the COVID-19 pandemic and had a consistent history of PA. Moreover, their average sedentary time was 7.95 h per day, substantially lower than the typical 9.4 h per day reported among community-dwelling older adults (6). Compared with studies conducted during the pandemic, the difference is even more pronounced, as older adults were found to accumulate approximately 11.38 h of sedentary time per day (34). Such comparatively lower SB levels in our sample may have attenuated the expected negative association with PP. Despite these results, we emphasize the importance of adopting strategies to reduce SB, based on previous findings showing that lower sedentary time is associated with better PP, particularly in lower and upper limb strength (3).

A sensitivity analysis was also conducted to investigate the effect of gender on PP variables, daily step count, and sedentary time. As expected, we observed a significant difference of approximately 10.7 kgF in the handgrip strength test. Men demonstrated higher handgrip strength than women, which is explained by physiological factors such as muscle mass, hormonal composition, and age-related declines (35). Regarding differences in sedentary time, women spent approximately 120 min less in sedentary activities than men. A review study also found that men tend to spend more time in sedentary activities, regardless of whether it is measured by self-report or objective instruments. One explanation for these findings is that women are more likely to engage in organized and group activities, perform more domestic, gardening, and recreational tasks, and have greater family and caregiving responsibilities than men (36).

This study had some limitations that should be taken into consideration. Firstly, we had a small sample size, which reduces statistical power and hinders generalizability of the results. However, the pandemic period was a limiting factor, especially for older adults, and therefore, we recommend conducting studies with a larger sample size and data on SB and PA collected during the pandemic. The low percentage of older men in the study is also a limitation. One possible explanation for this phenomenon is the high participation of older women in exercise programs and the preference of older men for outdoor and solitary activities. Men also tend to consider group programs as activities more oriented toward women (37-39). Another limitation is the cross-sectional design of the study, which does not allow us to infer causality between exposure and outcome. Including pre-pandemic data could be important for identifying longitudinal effects of sedentary time and daily step count on PP. Longitudinal studies should be conducted to identify the relationship between exposure and outcomes over time. The results of this study should also be considered with caution, as our sample consisted of Brazilian older adults with a history of PA who were enrolled in an exercise program prior to the COVID-19 pandemic, which may increase the likelihood of a potential selection bias. Therefore, we emphasize the need for future studies involving diverse populations, with data collected at different stages of the COVID-19 pandemic, conducted in other regions of Brazil and worldwide, and including older adults from various backgrounds and contexts.

## Conclusions

Daily step count was the only variable associated with PP of older adults during the COVID-19 pandemic. No associations were observed between SB and PP. Each additional 1,000 steps/day was positively associated with the maintenance of lower limb strength, handgrip strength, and functional mobility. Furthermore, older adults who accumulated approximately 9,300 steps/day exhibited better PP compared to those with lower daily step counts. In summary, accumulating a high number of daily steps, even during public health crises such as the COVID-19 pandemic, may contribute to the maintenance and improvement of PP among community-dwelling older adults.

## Data Availability

The datasets generated and/or analyzed during the current study are available from the corresponding author on reasonable request.
